# The Maryland Medical and Surgical Journal, and Official Organ of the Medical Department of the Army and Navy of the United States

**Published:** 1840-07

**Authors:** 


### BIBLIOGRAPHICAL.

*The Maryland Medical and Surgical Journal, and official organ of the Medical department of the army and navy of the United States.*

In a former number of our journal, we took occasion to notice the prospectus of the above work, and we are now happy to state, that the first number is fully equal to the high estimate we were pleased to place upon its character, in anticipation of its appearance. We were satisfied upon the announcement that was made of the gentlemen composing the editorial committee, that they would furnish the medical profession with a periodical every way worthy of encouragement, and we have not been disappointed.

The number before us opens with the address of the editors, in which their views and purposes are clearly expressed, and without that effort at display which is prominent in many of the publications of the day.

The memoir of the late Dr. Baker, speaks in high terms of that esteemed and talented gentleman ; but of his worth and character, the half is not said. In the character of professor Baker, there was every thing to imitate ; and long will his memory be cherished by the inhabitants of Baltimore.

Following the memoir, is a lecture on scarlatina, delivered by professor Baker about a year before his death, at the request of the Medico-Chirurgical Society of Baltimore. This is an able and interesting paper, and the editors have done well to insert it in the first number of their journal.

The use of the immovable apparatus in the treatment of fracture, though of recent adoption, is beginning to attract considerable attention. The number before us contains two interesting papers on this subject ; one by professor Jameson, and one by A. F. Dulin, M. D., both of Baltimore.

Dr. G. C. M. Roberts, in his *Obstetric* Memoranda has given several curious and interesting cases of ossified placenta ;—one, if we mistake not, unlike any on medical record. In this, it would seem that vital process of the fœtus from some unknown cause, was arrested, as we are told by Dr. R. about the forty-fifth day after conception, and in which state that, together with the secundines remained in utero until the expiration of nine months when it was expelled.

The article by Dr. T. H. Buckler of Baltimore, on the use of gold dust and iron filings as a galvanic antidote to corrosive sublimate and other poisonous compounds of mercury, will be read with interest, and may lead to valuable practical results. The author, in a note annexed to this article, passes a very deserved compliment on professor W. R. Fisher, long a resident of Baltimore, and who on account of continued ill health, was compelled to leave it. While professor of chemistry in the University of Maryland, he labored with the greatest assiduity, and his zeal in the cause of his profession had well nigh made a martyr of him in the "midst of his years." We would hope for this son of science, that he may find a more propitious clime, and that his wasted health may be renewed, so that he may yet win other laurels wherewith to crown his declining years.

[BALT. ED.

